# 3D-Culture System for Heart Regeneration and Cardiac Medicine

**DOI:** 10.1155/2013/895967

**Published:** 2013-09-08

**Authors:** Nanako Kawaguchi, Kota Hatta, Toshio Nakanishi

**Affiliations:** ^1^Department of Pediatric Cardiology, Tokyo Women's Medical University, 8-1 Kawada-cho, Shinjuku, Tokyo 162-8666, Japan; ^2^University of Toronto and University Health Network, Toronto, ON, Canada M5G 1L7

## Abstract

3D cultures have gained attention in the field of regenerative medicine for their usefulness as *in vitro* model of solid tissues. Bottom-up technology to generate artificial tissues or organs is prospective and an attractive approach that will expand as the field of regenerative medicine becomes more translational. We have characterized c-kit positive cardiac stem cells after long-term cultures and established a 3D-nanoculture system using collagen scaffolds. By combining informatics-based studies, including proteomic analyses and microarrays, we sought to generate methods that modeled cardiac regeneration which can ultimately be used to build artificial hearts. Here, we describe the use of biodegradable beads or 3D cultures to study cardiac regeneration. We summarize recent work that demonstrates that, by using a combination of molecular analyses with 3D cultures, it is possible to evaluate concise mechanisms of solid tissue stem cell biology.

## 1. Introduction

Heart failure is the leading cause of death in the Western world and is rapidly increasing worldwide [1–3], and it occurs due to the dysfunction or loss of cardiomyocytes [4]. Therefore, the generation of stem cell-derived cardiomyocytes has been an active area of research. In this regard, induced pluripotent stem (iPS) cells have been considered as therapeutic agents for their ability to differentiate into many cell types including cardiomyocytes [5, 6]. Indeed, methods for cardiomyocyte differentiation from iPS cells and the selection of mature iPS-derived cardiomyocytes have been established [7–9], and these iPS-derived cardiomyocytes were found to be overall similar to native tissue-derived cardiomyocytes [10]. On the other hand, Beltrami et al. in 2003 isolated and characterized tissue-resident cardiac stem cells (CSC). It was reported that these resident stem cells were also capable of contributing to the regeneration of the heart [11]. Thus, both iPS cells and tissue resident CSCs are targets for therapy. 

Recently, in our own work we have also isolated and characterized c-kit positive CSCs from adult hearts [12, 13]. In our studies, the characteristics of these cells varied between isolations after long-term culture [12]. Nonetheless, CSCs have the potential to be used for translational applications. For example, among the CSCs we isolated, CSC4A had less sphere-forming ability but higher cardiomyocyte differentiation ability [12, 14]. Furthermore, when cocultured, these cells enhanced cardiomyocyte survival *in vitro *via a paracrine mechanism. The effect was paracrine given that the increase in cardiomyocyte survival was observed even when cells were separated by 0.22 *μ*m pore-sized filter inserts in which solutions can pass, but cells cannot. 

iPS cells may be the most appropriate cell type for studying 3D cardiomyocyte cultures. The ability of iPS cells to differentiate into mature, functional, and beating cardiomyocytes is as good as embryonic stem (ES) cells [15–17]. However, when iPS- or ES-derived cardiomyocytes were compared to the native cardiac tissue, contractile properties were significantly different even when cells and tissues were of comparable age [16]. The difference could be attributed to the cell-line-dependent differentiation process that was reported in iPS cell-derived cardiomyocytes [18]. Nevertheless, the use of iPS cells as agents in translational applications has its advantages. (1) iPS cells can be autograft, meaning no immunorejection and no ethical problems such as those encountered with ES cells. (2) iPS cell banks are now being developed. As the iPS cell bank expands and becomes well stocked, their availability will be better than other types of stem cells. Human cells have different types of major histocompatibility complex antigens (human leukocyte antigen, HLA) on their cell surface. In therapy, if there is a mismatch in antigen type, transplanted cells would be recognized by the recipient as foreign cells and trigger immunorejection. iPS cell banks are being expanded to cover 170 different HLA genotypes which, practically speaking, allows for an immuno-compatible match for 80% of the Japanese population [19]. Therefore, there is a great incentive to use iPS cells as a foundation to develop 3D-culture systems that can eventually lead to translational applications. 

Alternative approaches of therapy are methods designed to enhance tissue-resident stem cells of the heart. The expansion and differentiation of cardiomyocytes from resident stem cells would restore cell loss and promote healing in the aftermath of a myocardial infarction. To promote resident stem cell function, microbeads embedded with growth factor can be transplanted in the infarcted area. Slow-release microbeads can be designed with optimal kinetics to concentrate the growth factors to the local site of injury. The ability to concentrate growth factors to a local area over an extended period of time makes bead-embedded delivery preferable over simply injecting growth factors alone. In considering what growth factors induce cardiac stem cells to differentiate, we found that TGF-*β* enhanced myogenesis and noggin induced beating cardiomyocytes from left atrium-derived pluripotent cells (LA-PCs) [20]. However, TGF-*β* can function to induce fibrosis by endothelial-mesenchymal transition (EMT). Therefore, the concentration of TGF-*β* should be controlled in a certain range and additional work on this question is required.

In this review, we summarize 3D *in vitro* culture methods and their possible application for the future of regenerative medicine and *in vitro* models for drug discovery.

## 2. Manufacturing a 3D System for Cardiac Tissue Regeneration

**Figure 1:**
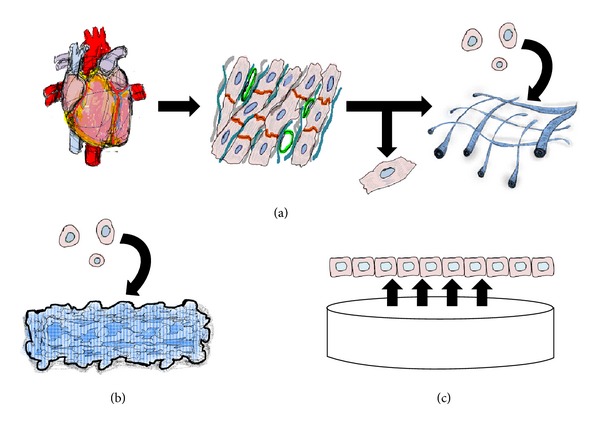
Two methods of making cardiac tissues. Decellularized hearts are seeded with cardiac cells (a). The seeded cells embed themselves in the matrix framework of the decellularized organ and reestablish cardiac tissue. Alternatively, bottom-up technologies are used to connect small pieces of tissues produced *in vitro *to reconstruct cardiac tissue artificially. The small pieces used for reconstruction could be regenerated tissues produced from scaffolds, hydrogels, cell sheets, or a combination of such materials. Shown here is an example of cardiac stem cells seeded on hydrogels (b) and cell sheets lifting off of a temperature responsive culture dish (c).

When considering the framework for growing cardiomyocytes into tissues-like structures for transplantation, there are three major types of materials for building cardiac tissues: collagen scaffolds, hydrogels, and cell sheets. Of note, scaffolds and hydrogels need to be fabricated prior to cell seeding and transplantation. The three materials are briefly summarized (Figure 1).

### 2.1. Extracellular Matrix (ECM) Scaffolds

Using scaffolds for cardiac regenerative medicine has a rather long history. In 2004, beating cardiac tissues were made using collagen scaffolds and neonatal cardiomyocytes with electrical stimulation [21]. It was a rather surprising report that a beating heart could be regenerated using decellularized (meaning decellularized enzymatically by perfusion) rat hearts that were subsequently injected with neonatal cardiomyocytes and endothelial cells [22]. This report suggested that if we can prepare the collagen frame and embed cardiomyocytes and endothelial cells, we can generate a beating heart. However, because of its size, there are practical limitations to using a similar technique to create a human heart. Considering that decellularized hearts have a suitable environment for cells and appropriate niches, a combination with synthetic materials [23] or development of more naturally decellularized methods may overcome this limitation [24].

Recent advances in 3D printing, a technology that allows for the construction of 3D scaffolds, are promising as computer-designed scaffolds can now be created using natural ECM rather than hydrogel and other polymers. Custom-designed scaffolds using 3D printing will in no doubt be exploited as tissue engineers determine optimal structures for cell seeding and tissue function in future research. As biological materials are best suited for such applications, the current understanding is that natural ECMs are the most appropriate. Injectable materials have been tested for their efficacy in treating postmyocardial infarction injury. Materials used include ECM prepared from decellularized hearts that were delivered via a catheter [25–27]. ECM prepared from the heart is a logical choice for injection into the heart. By extension, ECM prepared from decellularized cardiac tissue could be isolated, prepared into a powder, and fabricated into scaffolds as injectable materials or prepared for cell seeding. Indeed, such composite scaffolds have been shown to prevent left ventricular wall thinning postmyocardial infarction injury [28].

Scaffolds are not necessarily composed solely of collagen. Scaffolds can be customized by the addition (embedding or conjugation) of growth factors to promote tissue regeneration. Of note, mesenchymal progenitor cells (MPCs) prepared with decellularized matrix embedded with TGF-*β* had increased expression of alpha-smooth muscle actin (SMA), myocardin, and connexin 43 compared to controls [29]. The effect was variable based on the concentration of TGF-*β*. High concentrations of TGF-*β* upregulated osteogenesis (determined by the expression of osteogenic genes such as osteopontin, type X collagen, and aggrecan). However, low concentrations of TGF-*β* upregulated vasculogenic genes. Similar results were obtained using fibrin hydrogel. These TGF-*β* preconditioned hydrogels enhanced vascular network formation, probably by a mechanism involving the secretion of paracrine factors. Thus, scaffolds can additionally be customized by the addition of growth factors to direct the differentiation or the regenerative capacity of such biomaterials.

Collagen scaffolds can be created in various shapes. Novel architectures include the fabrication of collagen in a cylindrical shape (3D) and seeding chick embryonic cardiac cells. Contractile profiles of embryonic cardiac cells in this scaffold were investigated, and results suggested that the scaffold helped cells retain properties of the developing embryonic myocardium [30]. Indeed, maturation of cardiomyocytes seems to be affected by local tissue architecture. Microarray and phenotypic comparisons were made between cardiac cells cultured in 2D and 3D conditions [31]. Maturation markers and responsiveness to hormonal stimulation were more readily observed in 3D cultures, suggesting that conventional 2D cultures are limited in their applicability for study or translational potential. Additionally, 3D scaffolds with human fibroblasts have been tested in myocardial infarction models, and it was reported that treatment supported angiogenesis and healing [32]. 

In our own work, we recently established scaffolds [33] using CSCs and our sphere-forming CSC-21E cells [12, 34]. In the study, we compared cell proliferation in 2D, 3D, and 3D bioreactor cultures. As we expected, 3D bioreactor cultures activated cell proliferation the most. Thus, in addition to the presence of a scaffold, the culture vessel itself can affect the quality of cells harvested. 

### 2.2. Hydrogels and Synthetic Biodegradable Materials

Hydrogel fabrication protocols have been developed and are described elsewhere [35, 36]. The merit of using hydrogels is that they can be microfabricated by lithography to create customized patterns. A material commonly used for this is polyethylene glycol (PEG). PEG hydrogels are biologically inert, yet when injected into the left ventricular wall, the structural reinforcement they provide is insufficient to prevent negative left ventricular remodeling after myocardial infarction [36]. In a separate study a pH sensitive, temperature responsive synthetic polymer alone increased infarct wall thickness initially but slowly declined, indicating polymer injection alone does not improve cardiac function [37]. Improving postinjury cardiac function using hydrogels can be accomplished by combining hydrogels with cell agents. Indeed, in such experiments combining hydrogels with cells led to increased neovascularization, restoring the blood supply to the infarcted area [37–40].

Bottom-up technology, fabricating from small molecules a complete tissue, is attractive because the built tissue can be functional and suitable as *in vitro* models that can more closely model diseases. 

### 2.3. Cell Sheets

Cell sheets, as their name suggests, are thin layer(s) of cells connected to each other in a flat, sheet-like preparation. Cell sheets are created from 2D cultures prepared on temperature-responsive dishes (UpCell; CellSeed, Tokyo, Japan) [41, 42]. Cultures are expanded to confluency and lifted from the dish substrate by lowering the temperature to 20°C for 2 hours. UpCell culture dishes have a cell surface covalently grafted with a temperature-responsive polymer, poly(N-isopropylacrylamide), which are hydrophobic, so cells can adhere and proliferate at 37°C. However, they become hydrophilic by lowering the temperature below 32°C, so cells cannot adhere to the surface [43–46]. Therefore, there is no need to use digestive enzymes, such as trypsin, which can damage the cells. Cell sheets can be attached on top of damaged areas of tissue to provide physical protection or paracrine support. Layers of cell sheets have the potential to create thicker structures that could be transplanted to provide functional support of damaged tissues. In the future, we may see the technique be used to overlay cardiomyocyte cell sheets and with an embedded vascular system to, in essence, create an artificial heart [41, 42]. 

## 3. Proteome Analysis

Although numerous publications exist on the topic of proteomic analysis, little is known regarding protein expression differences between 2D and 3D cultures. Stem cells are known to form spherical (i.e., 3D) embryonic bodies (EBs). To compare 2D and 3D states, we recently compared the proteomic profiles of sphere-forming and adherent cells using CSC-21E [34, 47]. In this experiment, the same culture medium was used; however the dish substrate was changed. Using conventional cell culture dishes, CSC-21E cells were attached and normal fibroblastic morphology was observed. On the other hand, CSC-21E cells grown on bacterial dishes aggregated and formed spheres. Comparing 2D and 3D cultures, we found that most of the detected proteins were not differentially expressed. Nonetheless, several proteins were significantly different. From our analysis, chaperon proteins tended to overall be upregulated in the attached state. Additionally, we also found that annexin A6 was upregulated in 3D sphere-forming cultures but annexin A7 was down-regulated in 2D adherent cultures, suggesting that these molecules can be the switched in expression between spherical and adherent conditions. It was also interesting that the expression of embryonic genes was upregulated in the 3D cultures [48]. Finally, in 3D spherical cultures, anti-inflammatory proteins were also altered [49]. Thus, it is evident that 2D and 3D cultures, have different proteomic profiles. Proteomic analysis is a comprehensive method to compare 2D and 3D cultures and we believe the proteins described here could be used as markers for the evaluation of 3D cultures.

## 4. Microarray Analysis Comparing Undifferentiated and Differentiated Cells

LA-PCs become immature cardiac/skeletal myocytes or adipocytes when grown in MethoCult methylcellulose medium. We took interest in this phenomenon and performed microarray analysis to determine if there were specific signals that acted as molecular switches to direct LA-PC differentiation into myocytes or adipocytes. Interestingly, pathway analyses screens flagged members of the TGF-*β* signaling pathway as differentially expressed. As a followup, we performed additional cultures with TGF-*β* supplementation and observed a dose-dependent up-regulation of skeletal myogenesis and downregulation of adipogenesis [20]. Furthermore, the addition of noggin resulted in the downregulation of adipogenesis and produced high yields of beating cardiomyocytes. Further discussion regarding the results of this report has been described elsewhere [50, 51]. Dissection of the *in vivo* function of noggin and/or TGF-*β* is an ongoing area of research. Knowing which defined factors guide the differentiation of a specific desired effector cell would be useful. For example, growth factor embedded scaffolds or biomaterials are seeded with stem cells and transplanted. Alternatively, stimulating local, tissue-resident stem cells to differentiate into specified lineages guided by transplanted growth factor conjugated microbeads could be another strategy in tissue regeneration. 

## 5. Modeling Diseases *In Vitro* Using 3D Cultures

iPS cells can contribute not only to regenerative medicine but also to the fields of drug discovery and testing. Currently, the applicability of iPS cells in drug discovery has gained more attention than their potential use in tissue regeneration. iPS cells derived from patients or iPS cells engineered with a disease gene are differentiated into myocytes and are now being used to model diseases *in vitro* [52]. At present, only channelopathies, such as LQTS-type 2 [53] and LQTS-type 3 [54], have been well studied because of the comparatively easy functional analysis needed for characterization [51]. However, more complicated diseases will require culture methods that more closely model the native tissue environment. Therefore, 3D-culture models that resemble native tissues can be useful in working towards a greater understanding of diseases. For the purpose of regenerative medicine, future 3D-culture models that are heterogeneous in cell composition which include not only cardiomyocytes but also endothelial cells and smooth muscle cells should be developed for a more complete 3D model of cardiac tissue.

## 6. Conclusion

In summary, 3D-culture systems have several advantages over traditional 2D cultures. A 3D architecture in culture more closely mimics the native environment of the tissue. Cells grown in 3D cultures proliferate rapidly. When progenitor cells are grown in 3D, stemness can be modulated by manipulating the growth factors they are exposed to and direct differentiation towards a desired linage. Additionally, 3D cultures can be customized. Physical, chemical, and biological properties of a scaffold or vessel can be manipulated to manufacture unique materials to suit various purposes. 3D cultures can be used to study basic properties of stem cells, of diseases modeled by iPS cells, or contribute themselves as therapeutic agents. The comparative studies using 2D versus 3D cultures summarized here suggest that cells respond in entirely different ways to each system. The difference between these cultures indicates that additional molecular studies using 3D cultures may open new opportunities for future studies.
